# Development of *Pinaceae* and *Cupressaceae* Essential Oils from Forest Waste in South Korea

**DOI:** 10.3390/plants12193409

**Published:** 2023-09-27

**Authors:** Chanjoo Park, Heesung Woo, Mi-Jin Park

**Affiliations:** 1 College of Forest and Environmental Sciences, Kangwon National University, Chuncheon 24341, Republic of Korea; whs1608@kangwon.ac.kr; 2 Forest Industrial Materials Division, Forest Products and Industry Department, National Institute of Forest Science, Seoul 02455, Republic of Korea; lionpmj@korea.kr

**Keywords:** essential oils, unused forest biomass, *Pinaceae*, *Cupressaceae*, bioactive compounds

## Abstract

The growing awareness of environmental issues has garnered increasing interest in the use of waste material in a wide variety of applications. From this viewpoint, developing essential oils from forest waste can bring new cost opportunities for the effective and sustainable management of unused forestry biomass. However, better knowledge of the production, chemical constituents, and application of essential oils is necessary. Among the species considered to be of interest from the point of view of their essential oils and forest biomass, *Pinaceae* and *Cupressaceae* were selected in this study as potential candidates for commercial essential oils based on previous studies. This current study focuses on investigating *Pinaceae* (*Larix kaempferi*, *Pinus densiflora*, and *Pinus koraiensis*) and *Cupressaceae* (*Chamaecyparis obtusa* and *Chamaecyparis pisifera*) essential oils extracted from various parts from the perspective of their bioactive compounds and potential applications. This is followed by an overview of the essential oils industry in South Korea, with particular attention being paid to utilising unused forest biomass. Therefore, this is a comprehensive review suggesting that *Pinaceae* and *Cupressaceae* essential oils extracted from various parts of forest waste could be utilised in various industries, adding value to the aspect of sustainable industry. Furthermore, our study contributes towards capturing the value of forest resources through the utilisation of native essential oils in South Korea.

## 1. Introduction

There is a large and sustainable supply of foliage, twigs, bark, and wood residue, and there are notable value-adding opportunities for unattended forest waste [1]. Essential oils extracted from forestry biomass waste are valuable owing to their bioactive compounds [2]. However, a considerable amount of valuable biomass resources after forest management are still underutilised. This is still the case today in South Korea.

The purpose of our previous study was to screen potential commercial essential oils from forestry resources in South Korea. Specifically, we reviewed prospective candidates for native essential oils from the Essential Oils Bank, which was established by the National Institute of Forest Science (NiFoS) in terms of both forest management and conservation. From a total of 39 listed candidates from forest resources, *Pinaceae* (*L. kaempferi*, *P. densiflora,* and *P. koraiensis*) and *Cupressaceae* (*C. obtusa* and *C. pisifera*) could be potential candidates for producing commercial essential oils, as their waste materials are easily obtained from plantations after forest management [3]. In this study, we focus on the development of the selected native essential oils (*Pinaceae* and *Cupressaceae*) from the previous study by exploring further information about their biological activities and potential uses. 

The market for medicinal and aromatic plants is continuously growing, owing to the demand for their bioactive compounds and diverse uses [4]. Considering this situation, the high and increasing market value of essential oils could allow for the exploitation of forest resources to be economically viable [5]. Some studies have already been conducted to establish their better utilisation by converting under-valued waste resources. Specifically, essential oils and hydrolate (*Cupressus lusitanica* and *Cistus ladanifer*) from biomass wastes showed strong anti-inflammatory properties [2]. Hence, increasing the applications and markets for essential oils could bring new opportunities for the cost-effective, profitable, and sustainable management of unused forestry biomass. However, better knowledge of the biological activities and various applications of targeted essential oils is necessary for the development of essential oils from forest waste in South Korea. Therefore, this is a comprehensive review suggesting that forest waste could be utilised as a source of essential oils from the perspective of recently reported work in this field, together with the current status of the forestry and essential oils industry in South Korea. Specifically, we review the targeted essential oils, paying particular attention to their bioactive compounds and various applications.

## 2. Essential Oils Industry in South Korea

At present, there is a growing interest in essential oils, owing to their bioactive compounds together with their pleasant fragrance. Djilani and Dicko [6] explained that approximately 300 essential oils have been produced from at least 2000 plant species, out of which 300 are essential commercial crops. Table 1 shows the quantities of major commercial essential oils produced worldwide.

In South Korea, the domestic essential oil market heavily relies on imports from overseas. According to the trade statistics provided by the Korea Customs Service, the trade in essential oils and resionoids (H.S. code: 33) has resulted in a trade deficit (Table 2). There are several reasons for this.

First, the level of industrial essential oil technology remains at a fairly basic level by processing just the formulation and dilution. Although there are a variety of native aromatic crops in South Korea, it is still challenging to develop high-quality essential oils or fragrance products at the industrial level, owing to a lack of professional skill and expertise [9,10]. Second, the cultivation area and production volume of aromatic and medicinal crops are very limited in South Korea. So, there would be low price competitiveness compared to foreign products in the global market. Third, most studies of native aromatic and medicinal plants focused on the investigation of biological activities for developing herbal medicine from plant extract [11]. On the other hand, there is limited research on the development of native essential oils, including but not limited to production, extraction, and standardisation. The current study simply focused on the identification of chemical components and biological activities in oils. [12,13]. Due to these reasons, it is problematic to develop the commercial essential oil industry in South Korea. However, it is indispensable to discover and develop commercial essential oils from native plants in South Korea in response to Nagoya’s protocol.

## 3. Investigating the Selected *Pinaceae* and *Cupressaceae* for Essential Oils

Essential oils are highly volatile in the air, and their fragrances vary depending on plant material and species [14]. Specifically, the essential oils are obtained from flowers, buds, seeds, leaves, bark, fruits, and roots [15]. Yet, there is limited information on the chemical profile and scent descriptions of essential oils extracted from various parts. Based on the Essential Oils Bank, which was established by the National Institute of Forest Sciences (NiFoS), the fragrance information of *Pinaceae* and *Cupressaceae* was only focused on the essential oils extracted from leaves.

We focus on screening the *Pinaceae* and *Cupressaceae* essential oils from various parts from the perspective of their bioactive compounds and various applications. However, the chemical profile can be variable, even within the same species. Specifically, the variation in the chemical components of essential oils can be caused by genetic and environmental factors [16]. Furthermore, biological activity can be highly variable in terms of bioactive components in oils. Hence, this section is written with the purpose of giving an overview of current knowledge about the chemical profile and biological activity of targeted essential oils so as to find research areas that can facilitate the applications of essential oils in various industries.

### 3.1. Pinaceae Essential Oils

#### 3.1.1. *L. kaempferi*

*L. kaempferi* is one of the main coniferous species in South Korea, so it is a major contributor to the national forest stock [17,18]. Because of this, most studies have been focused on the optimisation of plantations for efficient forest management [19,20,21], and there are limited studies on the extract and essential oils from *L. kaempferi*. The overall information on the essential oils extracted from the leaves and wood is summarised in Table 3. The essential oils from the leaves demonstrated herbicidal activity. According to the greenhouse experiment, treated plants by foliar application of 10% essential oils showed a burndown effect on their leaves [22]. Furthermore, Kim et al. [23] found that *L. kaempferi* essential oils from leaves showed an anti-dermatophyte effect, owing to the active antifungal components, such as terpinene-4-ol, α-terpineol, and α-cadinol. Lastly, the essential oils extracted from wood could be used as an anti-inflammatory agent [24].

#### 3.1.2. *P. densiflora*

*P. densiflora* has been used in folk medicine for a long time in South Korea. Pine needles have been widely used as food ingredients, food additives, and folk medicines in pine-based products due to their characteristic aroma, taste, and health [25,26]. For example, the leaves of *P. densiflora* have been used to prepare drinks as tea, and pine needles have been used in folk medicines for liver diseases, skin diseases, and hypertension [27,28,29]. Currently, *P. densiflora* extract is being included in cosmetic and functional foods and sold in the local market in South Korea. As shown in Table 4, the essential oils are extracted from the leaves, wood, and twigs of *P. densiflora.* Furthermore, their major chemical components, biological activities, and potential uses of essential oils extracted from various parts are listed, respectively. Sangwan et al. [16] mentioned that the chemical profile can vary depending on the specific part used from the same plant. The *P. densiflora* essential oils’ chemical components in leaves and twigs are similar. However, the component α-pinene is the most abundant in leaf essential oils, while β-pinene is predominantly found in the essential oils extracted from twigs [30]. Unlike essential oils from the leaves and twigs of *P. densiflora*, the major chemical component of wood essential oils is longifolene (14.3%) [24].

*P. densiflora* essential oils extracted from various parts show diverse biological activities. *P. densiflora* leaf essential oils showed antioxidant and anti-ageing activity owing to phellandrene and *B*-pinene. So, it could be used as a functional cosmetic ingredient for anti-wrinkle benefits [30]. Furthermore, essential oils contain anti-proliferative, anti-survival, and pro-apoptotic effects on human oral squamous cells because of α-pinene, leading to the cytotoxic effect [31]. Hence, it could have potential usefulness in cancer prevention. Lastly, essential oils show strong antibacterial activities [32]. The essential oils can be used as natural antimicrobial and antibacterial substances in the food industry and the household cleaning product industry. The essential oils from wood contained the chemical components of α-pinene (47.2%), longifolene (14.3%), and β-phellandrene (11.8%). The essential oils with bioactive compounds such as β-pinene and longifolene showed an anti-inflammatory effect by inhibiting the degranulation and expression of cytokines [24]. Hence, it could be conducive to relieving allergic inflammation, which can be used for pharmaceutical purposes. Lastly, essential oils from twigs of *P. densiflora* also showed antioxidant and anti-ageing activities in terms of similar chemical constituents in essential oils extracted from leaves [30].

**Table 4:** The major constituents, biological activities, and potential application of essential oils extracted from *P. densiflora*.

Scientific Names	Plant Parts	Major Chemical Profile	Biological Activities	Application	Ref.
*P. densiflora*	leaves	α-pinene (21.6%) limonene (13.1%) caryophellene (11.4%) [30]	antioxidant and anti-ageing activities	cosmetic industry	[30]
			anti-cancer	pharmaceutical industry	[31]
			antibacterial	food industry	[32]
	wood	α-pinene (47.2%) longifolene (14.3%) β-phellandrene (11.8%)	anti-inflammatory effect	pharmaceutical industry	[24]
	twigs	β-pinene (22.4%) α-pinene (17.3%) limonene (15%) [30]	antioxidant and anti-ageing activities	cosmetic industry	[30]

#### 3.1.3. *P. koraiensis*

*P. koraiensis,* commonly called Korean nut pine, has been used as a food supplement and in traditional Asian medicine for the longest time [33]. According to Table 5, *P. koraiensis* essential oils are extracted from seed, wood, cones, and leaves. The major constituents of EO obtained from the seed and cone are α-pinene (29.9%), D-limonene (19.3%), and *Β*-pinene (11.2%). The essential oils showed mild antimicrobial properties by inhibiting the growth of other pathogens related to acne. Therefore, it can be used as a natural cosmetic ingredient or as an antimicrobial substance [14].

The essential oils from wood showed an anti-inflammatory effect. In particular, the chemical components of *Β*-pinene and α-terpineol in oils were assumed to be the most contributing bioactive compounds to the anti-inflammatory activity [34].

Essential oils extracted from cones promoted anti-metastatic activity in breast cancer cells by inhibiting tumour necrosis through the action of bioactive compounds [35]. Moreover, the cone essential oils showed significant antimicrobial activities, especially against pathogenic fungal strains such as *Candida glabrata* YFCC 062 and *Cryptococcus neoformans* B 42419. Therefore, the results indicate that the essential oils from the cones can be used in various ways as a non-toxic and environmentally friendly disinfectant [36].

Essential oils extracted from leaves show a variety of biological activities, and they can be used in various manners. First, essential oils extracted from leaves showed anti-cancer activity via the inhibition of PAK1 expression, suggesting they might be a potent chemotherapeutic agent for colorectal cancer [37]. Second, they showed the anti-diabetic effect in mice with streptozotocin (STZ)-induced type 1 diabetes and on HIT-T15 pancreatic B cells, employing the hypoglycaemic potential effects [38]. Therefore, it can be a good candidate for natural anti-diabetic materials. For example, hypoglycaemic herbal medicines could be used for long-term diabetes patients with less toxicity [39,40]. Furthermore, the essential oils from the leaves showed antifungal activity against *Candida albicans* [41]. Lastly, *P. koraiensis* essential oils have effective deodorisation and inhibitory activity against the oral cavity in this study and might be potential material in the oral sanitary industry [42].

**Table 5:** The major constituents, biological activities, and potential application of essential oils extracted from *P. koraiensis*.

Scientific Names	Plant Parts	Major Chemical Profile	Biological Activities	Application	Ref.
*P. koraiensis*	seed and cone	α-pinene (29.9%) D-limonene (19.3%) *Β*-pinene (11.2%) [14]	anti-microbial activity (acne)	cosmetic industry	[14]
	wood	α-pinene (27.0%) *Β*-pinene (11.2%) α-terpineol (7.1%)	anti-inflammatory effect	pharmaceutical industry	[34]
	cones	D-limonene (28.0%), α-pinene (23.9%), *Β*-pinene (12.1%) [41]	anti-cancer	pharmaceutical industry	[35]
			anti-microbial activity	sanitary industry (disinfectant)	[36]
	leaves	α-pinene (10.5%) myrcene (7.3%) bornyl acetate (7.2%) [41]	anti-cancer (colorectal cancer)	pharmaceutical industry	[37]
			anti-diabetic effect	pharmaceutical and	[38]
			antifungal effect	food industry	[41]
			anti-oral microbial activity and deodorisation effect	sanitary industry (dental)	[42]

### 3.2. Cupressaceae Essential Oils

#### 3.2.1. *C. obtusa* Oil

*C. obtusa* has been widely used for various household purposes in South Korea. In general, *C. obtusa* has been used to make furniture owing to the high quality of the timber [43]. Also, the oil extracted from the branches, leaves, and twigs of *C. obtusa* has been commercially used as a functional additive in the production of soap, toothpaste, and cosmetics owing to its unique fragrance and bioactive compounds [44]. Furthermore, inhalation with the essential oils of *C. obtusa* is known as forest bathing or aromatherapy [45].

As shown in Table 6, *C. obtusa* essential oils are extracted from leaves, wood (sawdust), and fruit. The major chemical components of oils differ depending on the plant part from which they are extracted. In particular, essential oils from the leaves have been widely studied for several biological activities. First, *C. obtusa* essential oils showed an antibacterial effect by inhibiting the growth of various microorganisms. Specifically, essential oils were found to be effective in inhibiting the growth of various airborne microorganisms in indoor spaces [46]. Musee et al. [47] emphasised that disinfectants should not only be effective in reducing the microbes present in the air but also not be toxic to humans. Also, essential oils showed a deodorisation effect by mitigating an offensive odour [48]. Therefore, essential oils could be used as safe natural disinfectants and deodorants in the household cleaning industry. Second, *C. obtusa* oil has potential as a functional cosmetic ingredient. It can be utilised in anti-ageing targeted products for wrinkles, skin-barrier, and moisturising effects [49] and supplemental products for improving atopic dermatitis [50]. Third, *C. obtusa* oil could be a novel source of inflammation-specific pharmacological drugs, especially for peripheral pain. Based on in vivo experiments (mice), the inflammatory effect of *C. obtusa* oils was elucidated [51,52]. Yet, further research is needed to investigate the specific mechanisms and potential side effects of *C. obtusa* essential oils [51]. Fourth, essential oils with bioactive compounds showed insecticidal activity. Owing to bornyl acetate and terpinyl acetate, essential oils showed insecticidal activity, implying their potential use as strong insecticides [53]. Park et al. [54] found that essential oils could be useful in food and agriculture by managing the populations of rice weevils. Also, it can be potentially used as a ‘human-friendly’ insect repellent [55]. Lastly, *C. obtusa* oils might promote hair growth in an animal model and show a positive regulator of hair growth, owing to the bioactive components cuminol, eucarvone, and calamenene [56]. Additionally, the essential oil extracted from the pruned leaves and twigs showed both anti-cariogenic and anti-inflammatory effects. Specifically, *C. obtusa* essential oil inhibited the decrease in pH induced by *Streptococcus. mutans*, thereby inhibiting dental caries [57].

Currently, research on *C. obtusa* essential oils from sawdust and fruit is lacking. By recycling wood waste, it can be transformed into commercially valuable products, such as essential oils [58]. The major constituents of *C. obtusa* essential oils from sawdust are juniper camphor (12.5%), fonenol (12.4%), and d-9-capnellene-3-b-ol-8-one (10.1%). It showed high antioxidant activity; therefore, it could be a natural antioxidant in food by stabilising food against oxidative deterioration [59]. The major essential oils from the fruit of *C. obtusa* include *B*-caryophyllene (23.7%), myrcene (8.1%), and *p*-cymene (7.6%) [60]. Yet, there has been no research on the identified biological activity and potential application of essential oils from fruit.

**Table 6:** The major constituents, biological activities, and potential application of essential oils extracted from *C. obtusa*.

Scientific Names	Plant Parts	Major Chemical Profile	Biological Activities	Application	Ref.
*C. obtusa*	leaves	α-terpinyl acetate (13.7%) sabinene (11.0%) isobornyl acetate (8.9%) [61]	antibacterial and antimicrobial effect	household cleaning industry (disinfectant and deodorant)	[46,48,62]
			anti-ageing effect	cosmetic industry	[49]
			relieving the allergy (atopic dermatitis)		[50]
			anti-inflammatory effect	pharmaceutical industry	[51]
			anti-nociceptive and anti-inflammatory effects		[52]
			insecticidal activities	agriculture and food industry	[54,55]
			hair growth	functional cosmetic industry	[56]
	leaves and twig	α-terpinene (40.6%) bornyl acetate (12.5%) α-pinene (11.4%) [57]	anti-cariogenic effect	pharmaceutical industry	[57]
	saw-dust	juniper camphor (12.5%) fonenol (12.4%) d-9-capnellene-3-b-ol-8-one (10.1%)	antioxidant activity	food industry	[59]
	fruit	*B*-caryophyllene (23.7%) myrcene (8.1%) *p*-cymene (7.6%) [60]	-	-	-

#### 3.2.2. *C. pisifera* Oil

Among the other selected essential oils, very little work has been done on the study of *C. pisifera* essential oils. The major components in *C. pisifera* essential oils extracted from leaves are 3-carene (35.0%), (−)-bornyl acetate (19.8%), and α-pinene (13.0%) [63] (Table 7). The *C. pisifera* essential oil showed strong insecticidal activity [64]. However, little work has been done on the essential oils extracted from the fruit. Hong et al. [60] studied the chemical profile of *C. pisifera* essential oils extracted from fruit. The major chemical components are (−)-3-carene (30.3%), α-pinene (29.4%), and myrcene (15.1%).

## 4. Utilising the Forest Waste after Forest Management for Essential Oils

Due to climate change, many countries have declared themselves Net-Zero countries, including South Korea. For example, in 2018, the National Institute of Forest Science (NiFoS) was established to promote an efficient utilisation system for forest resources in renewable energy supply through the use of forest biomass waste.

Since 1972, South Korea has conducted a periodic National Forest Inventory (NFI) to manage forest resources efficiently and provide fundamental public data for forest policy. There is an increasing demand for public data and statistics to assess the economic and social value of forests, such as greenhouse gas absorption and the maintenance and enhancement of biodiversity. The NFI is a 5-year periodic monitoring system, and the report consists of five sections, including statistics on forest area, timber volume, timber resources (number of trees, stand volume, large-sized timber, and quality grade), biomass, carbon storage, and greenhouse gas absorption in the forest sector [65].

According to Table 8, the selected species could ensure a constant supply from forest management. However, the cost of transportation of forest biomass from forest management would need to be considered. Cambero and Sowlati [66] emphasised that the viability and feasibility of utilising the forest biomass would depend on ensuring the long-term availability of biomass supply with the required quality at a competitive cost. Forest residues after forest management are scattered over wide regions, so the extra cost of collection, handling, and transportation would also need to be considered [67]. Essential oil production from wild trees depends essentially on the abundance of the species and the costs of harvesting. Yet, oil production from the cultivated species includes considerable investments in the plantations [68]. Essential oils from wild sources are generally commercialised in low volume and at high prices, which can ensure a profitable industry. Hence, developing an essential oil industry from forest biomass needs to be a cost-efficient part of the forest biomass supply chain.

Unused forest waste includes diseased trees caused by pine wilt disease in South Korea. The stressed or diseased plant material can develop larger amounts of volatiles, and undesirable chemical compounds can create an allergic response. Figueiredo et al. [68] explained that stressed or diseased plant material induces increased amounts of volatiles, and undesirable chemical compounds can create an allergic response. The relationship between oil content and damage could be related to oil composition rather than oil yield, and this could differ within as well as between species. Therefore, further research is needed to identify the chemical profile of targeted essential oils extracted from unused forest biomass for safety efficacy. In conclusion, recycling and reuse of these renewable resources strengthen industries and technology to evolve in more sustainable ways and create more opportunities for the forest industry [69].

## 5. Safety of Essential Oils

Owing to their bioactive compounds and pleasant fragrance, essential oils have been used in various industries in recent times. Essential oils contain natural constituents, leading consumers to consider essential oils safe and nontoxic. Yet, the use of essential oils may still be hazardous for humans. For example, α-pinene is considered one of the allergenic chemical components in tea tree oil (*Melaleuca alternifolia* Cheel.), which is used all around the world [70]. In general, possible adverse effects of essential oils include nausea, vomiting, necrosis, mucous membranes, and skin irritation [71].

According to the European Union (EU) Cosmetics Regulation, 18 chemical components in essential oils contain potential allergen substances [72]. In particular, the most frequently encountered chemical components in oils are citral, citronellol, eugenol, farnesol, geraniol, limonene, and linalool. For safety reasons, the provider must be declared on the packaging or in the information description if the concentration of these allergenic fragrances is higher than the permissible concentration in the product [73].

The screened essential oils in this current study contained allergenic chemical components, such as limonene and D-limonene (Table 9). Both chemical components are widely used as fragrance and flavouring agents in cosmetic products, pharmaceuticals, perfumes, and food. As shown in Table 9, *P. densiflora* essential oil contains the allergenic chemical component limonene. When exposed to air, it can be easily oxidised to carvone, carveol, and limonene oxide. Altered chemical components can cause skin irritation and dermal sensitisation [72]. Several studies have reported that the oxidation products of limonene can cause allergic contact dermatitis [74,75]. The major component of *P. koraiensis* essential oil extracted from seed and cone is D-limonene. It is an irritant in high concentration, and its oxidation products, such as limonene oxide and limonene hydroperoxides, are allergenic, too [76,77]. For example, Roman chamomile (*Anthemis nobilis*) might show an allergic reaction owing to D-limonene [78]. Therefore, a more careful approach is needed when developing *P. densiflora* and *P. koraiensis* essential oils for use in cosmetics and pharmaceuticals, with respect to their suitability for tracking the chemical alternations in oils. Moreover, it is also necessary to develop reproducible and reliable analytical methods for assessing both pure and altered essential oils as part of quality control.

Chemical components in oils are known to easily covert into each other by oxidation, isomerisation, cyclisation, or dehydrogenation reactions, triggered either enzymatically or chemically [79]. The oxidation of essential oils would increase their allergenic potency. Rudbäck et al. [80] found that α-terpinene undergoes rapid autoxidation upon air exposure, and oxidation products can induce the contact allergy. Specifically, the sensitisation potency of autoxidised α-terpinene was nearly nine times higher compared to that of pure α-terpinene. Hence, it is important to use essential oils safely and carefully to minimise the potential adverse risks associated with the problematic and undesirable chemical constituents. Furthermore, it might be advisable for the industry to provide recommendable storage conditions and use-by dates for essential oils to ensure their stability and safety.

## 6. Limitations and Prospects

The shortage of landfill space, greenhouse gas emissions, and residue runoff have spurred efforts to find alternative ways for traditional waste management [5]. For example, new composites utilising organic waste and residues from agricultural and industrial processes have been developed [81]. Likewise, there is a case study to assess the opportunities, constraints, and information required to integrate the recovery of essential oils into forest and mill operations, as might be used in northern Arizona. Northern Arizona’s forest is composed of ponderosa pine (*Pinus ponderosa*), which produces timber, essential oils, and resins. The study confirmed the potential output with a programme of forest thinning and biomass utilisation [82]. In another case, there are several plant species (*Pinus pinaster*, *Eucalyptus globulus*, *Cistus ladanifer*, and *Lavandula* sp.) in the Algarve region (southern Portugal), which contribute to a significant amount of residual biomass after forest management. Lastly, there was collaborative governance in *Eucalyptus* oil industry development in forest areas in Indonesia. The study emphasised building a shared vision among the actors involved as a reference for success in the development of the *Eucalyptus* oil industry in the future. This would be available to achieve productive and sustainable forest management through collaboration among the government and the essential oil industry [83].

There is no current pilot-scale study for the targeted essential oil crops in South Korea, including but not limited to oil yield, chemical constituents, safety efficacy, and biological activity. However, the global essential oil market requires consistent, high-quality products with reliability of supply at competitive prices. This pilot-scale study would be crucial in evaluating the business potential for the development of the native essential oil industry in South Korea. Therefore, processing the pilot-scale study for the development of native essential oils is urgently needed. According to a pilot-scale study, essential oil yields were lower than those performed on a laboratory scale and reported in the literature. Furthermore, it is recommended to use industrial equipment for the production of essential oils. Processing at an industrial scale would allow optimisation of the operating conditions for commercial essential oil production [5]. Moreover, qualifying the International Organisation for Standardisation (ISO) would strengthen the market competitiveness of the essential oils domestically as well as internationally. For example, the chemical profile of tea tree oil (*Melaleuca alternifolia*) has been regulated by the ISO entitled “Oil of Melaleuca terpinen-4-ol type (tea tree oil)” (ISO-4730) and specifies the chemical compositional limit for the 14 constituents [84]. ISO standardised many analytical methods for controlling the quality of chemical components in oils, thus focusing on the quality control of essential oils in the global market [85].

To develop commercial native essential oils from forest waste in South Korea, it will be necessary to process further research under the pilot study levels below. Firstly, the mentioned potential candidates will require extraction of the oils and then confirmation of oil yield and chemical constituents. After that, the identified chemical constituents in oils would be able to investigate the various biological activities. It would be conducive to utilising essential oils based on identified bioactive constituents in oils. There are numerous studies on the biological activities of essential oils, elucidating their mechanisms [86]. In particular, studies on antimicrobial, antioxidant, anti-inflammatory, and anti-cancer activities have been investigated in many cell and animal models. However, very little work has been carried out on human clinical studies as phytotherapeutic agents, but the anticancer activity of essential oils should take a careful approach to the development of phytotherapeutic agents. Essential oils and aromatherapy are considered complementary therapies at this stage. Therefore, clinical studies are needed to investigate the real efficacy and safety of essential oils [87].

## 7. Conclusions

Unused forest biomass, a valuable renewable resource, has opportunities for sustainable energy production and other potential applications. Owing to the lack of professional skill and expertise, it will be challenging to develop a commercial essential oil industry in South Korea. However, there are clear imperatives to discover and develop essential oils from native plants in South Korea in response to Nagoya’s protocol.

A precedent study on the development of native essential oils from forestry resources suggested *Pinaceae* and *Cupressaceae* could be potential candidates for essential oils in terms of forest management and conservation in South Korea. By deepening the previous study, we reviewed the *Pinaceae* (*L. kaempferi*, *P. densiflora*, and *P. koraiensis*) and *Cupressaceae* (*C. obtusa* and *C. pisifera*) essential oils extracted from various parts from the perspective of their bioactive compounds and potential applications. The current study, along with the previous study, has confirmed the possibility of the idea that unused forest biomass may potentially be an untapped valuable source of essential oils. However, it is important to set up a cost-efficient system for the forest biomass supply chain.

By addressing the challenges and embracing the opportunities, *Pinaceae* and *Cupressaceae* essential oils extracted from various parts showed diverse biological activities. Therefore, essential oils of *Pinaceae* and *Cupressaceae* extracted from unused forest biomass could be used in various ways as non-toxic and environmentally friendly products.

## Figures and Tables

**Table 1:** Production figures of major commercial essential oils [7].

Essential Oils	Production Metric Tons	Main Production Countries
Orange oils	51,000	United States, Brazil, Argentina
Cornmint oil	32,000	India, China, Argentina
Lemon oils	9200	Argentina, Italy, Spain
Eucalyptus oils	4000	China, India, Australia, South Africa
Peppermint oil	3300	India, United States, China
Clove leaf oil	1800	Indonesia, Madagascar
*Citronella* oil	1800	China, Sri Lanka
Spearmint oils	1800	United States, China
Cedarwood oils	1650	United States, China
*Litsea cubeba* oil	1200	China
Patchouli oil	1200	Indonesia, India
Lavandin oil Grosso	1100	France

**Table 2:** The latest trade data on essential oils provided by trade statistics provided by the Korea Customs Service [8].

Period	Export Weight	Export Value	Import Weight	Import Value	Balance of Trade
2020	51.1	1809.6	3075	57,892	−54,817
2021	61.6	1831.9	2145	62,710	−60,565
2022	43.6	1650.0	1876	64,510	−62,634

Unit: USD/ton.

**Table 3:** The major constituents, biological activities, and potential application of essential oils extracted from *L. kaempferi*.

Scientific Names	Plant Parts	Major Chemical Profile	Biological Activities	Application	Ref.
*L. kaempferi*	leaves	α-pinene (19.9%), β-pinene (17.4%), L-bornyl acetate (6.1%) [22]	herbicidal effect	agriculture industry	[22]
			anti-dermatophyte effect	pharmaceutical industry	[23]
	wood	α-pinene (18.6%), α-cadinol (6.2%), cembrene (6.1%)	anti-inflammatory effect (relieving the allergy)	pharmaceutical industry	[24]

**Table 7:** The major constituents, biological activities, and potential application of essential oils extracted from *C. pisifera*.

Scientific Names	Plant Parts	Major Chemical Profile	Biological Activities	Application	Ref.
*C. pisifera*	leaves	3-carene (35.0%) (−)-bornyl acetate (19.8%) α-pinene (13.0%) [63]	insecticidal activity	agriculture	[64]
	fruit	(−)3-carene (30.3%) α-pinene (29.4%) myrcene (15.1%) [60]	-	-	-

**Table 8:** The forest area of targeted species in South Korea [65].

	Species	The Forest Area (ha)
1	*Larix kaempferi* (Lamb.) Carriere	259,257
2	*Pinus densiflora* Siebold & Zucc.	1,321,878
3	*Chamaecyparis obtusa* (Siebold & Zucc.)	69,538

**Table 9:** The allergenic chemical components in *P. densiflora* and *P. koraiensis* essential oils.

Scientific Names	Plant Parts	Major Chemical Profile	Allergenic Chemical Components in Accordance with EU Directive
*P. densiflora*	leaves	α-pinene (21.6%) limonene (13.1%) caryophellene (11.4%) [30]	limonene
	twigs	β-pinene (22.4%) α-pinene (17.3%) limonene (15%) [30]	limonene
*P. koraiensis*	seed	α-pinene (29.9%) D-limonene (19.3%) *Β*-pinene (11.2%) [14]	D-limonene
	cones	D-limonene (28.0%), α-pinene (23.9%), *Β*-pinene (12.1%) [42]	D-limonene

## Data Availability

Not applicable.
